# Therapeutic potential of *Lactococcus lactis* D4 from fermented buffalo milk (Dadiah) against *Staphylococcus aureus*-induced diarrhea in mice

**DOI:** 10.5455/javar.2026.m1039

**Published:** 2026-06-22

**Authors:** Ade Sukma, Azura Adawiya, Elsa Badriyya, Najmiatul Fitria

**Affiliations:** 1 Department of Livestock Product Technology, Faculty of Animal Sciences, Universitas Andalas, Kampus Unand Limau Manis, Padang 25163, West Sumatera, Indonesia; 2 Department of Pharmacology and Clinical Pharmacy, Faculty of Pharmacy, Universitas Andalas, Kampus Unand Limau Manis, Padang 25163, West Sumatera, Indonesia

**Keywords:** buffalo milk, diarrhea, *Lactococcus lactis* D4, mice, probiotics, SDG 3

## Abstract

**Objectives:** This study aimed to evaluate the therapeutic potential of *Lactococcus lactis* D4 against diarrhea induced by *Staphylococcus aureus* in a mouse model.

**Materials and Methods:** A randomized design was adopted using 25 male mice randomly assigned to five groups, which are negative control, positive control (diarrhea + cotrimoxazole), probiotic dose 1 (5 × 10^8^ CFU/mice in 0.5 ml), probiotic dose 2 (administered twice, each administration containing 5 × 10^8^ CFU/mice in 0.5 ml), and disease control (diarrhea without therapy). Diarrhea was induced with *S. aureus* (5 × 10^8^ CFU/mouse in 0.5 ml), and thereafter, the probiotic was given orally for 7 days. The main parameters examined were body weight, fecal weight, and consistency.

**Results:** Administration of *L. lactis* D4 improved diarrheal consequences (*p* < 0.05). The highest efficacy was observed at probiotic dose 2, containing 5 × 10^8^ CFU/mouse in 0.5 ml twice a day. This improvement made a little body weight loss (31.04 gm) and improved fecal consistency.

**Conclusions:** *Lactococcus lactis* D4 showed potential as an ecological probiotic for diarrheal therapy caused by *S. aureus*. These results supported the sustainable development goals (SDGs) of buffalo-derived functional foods to improve gut health (SDG 3) and informed sustainable strategies for global food security (SDGs 2, 9, and 12).

## 1. Introduction

Diarrhea remains a global health problem with high morbidity and mortality, especially among children in developing countries. The World Health Organization (WHO) reported approximately 1.7 billion cases of diarrhea every year. There are more than 400,000 deaths among children under five years old due to diarrhea [1]. Infective bacteria caused by *Staphylococcus aureus* are the major cause of diarrhea. *Staphylococcus aureus* is a Gram-positive bacterium that can lead to intestinal inflammation (healing and emptying the bowel) and disruption of the gut [2, 3, 4]. This bacteria can also invade the intestine and contribute to microbial dysbiosis [5]. The general remedy for diarrhea is antibiotics like cotrimoxazole and those tablets that have been commonly given by drug treatment. However, ongoing methicillin-resistant *S. aureus* (MRSA) strains in gastroenteritis have compromised conventional antibiotics such as cotrimoxazole [6]. This highlights the need for more sustainable alternative therapies.

Probiotics have become a promising new option for diarrhea treatment. Probiotics are live microorganisms that can have positive effects on the gut microbiota, producing antimicrobial compounds and fortifying the intestinal barrier [7, 8]. Dadiah, a traditional buffalo milk fermentation product from West Sumatra, is known to contain various lactic acid bacteria, such as *Lactococcus lactis* [9, 10, 11, 12]. The *L. lactis* D4 (LD4) strain isolated from Dadiah produces lactisin as the most stable bacteriocin for the digestive response. This is widely approved as an antimicrobial target for *S. aureus* [9, 13].

But the effects of this probiotic on diarrhea caused by *S. aureus* infection are very limited [10, 14]. In this study, we attempted to investigate how well the oral suspension of *L. lactis* D4 taken from Dadiah induces diarrhea in mice with *S. aureus* based on body weight, fecal consistency, and fecal weight to support Sustainable Development Goals (SDGs).

## 2. Materials and Methods

### 2.1. Ethical approval

This research complies with the ethical considerations of the Faculty of Pharmacy, Universitas Andalas, No. 77/UN16.10.D.KEPK-FF/2025.

### 2.2. Study design

This study employed a completely randomized design (CRD) with an experimental approach [3, 15], following Animal Research: Reporting of In Vivo Experiments (ARRIVE) guidelines from the Equator Network [3, 15].

### 2.3. Experimental animal

A total of 25 male mice (weighing 25–35 gm) were randomly assigned into five groups: Negative control: Healthy mice without diarrhea induction or therapy; positive control: Diarrhea + cotrimoxazole; treatment 1: Diarrhea + *L. lactis* D4 at a dose of 5 × 10^8^ CFU/mouse in 0.5 ml; treatment 2: Diarrhea + *L. lactis* D4 at a dose administered twice, each administration containing 5 × 10^8^ CFU/mouse in 0.5 ml; disease control: mice with diarrhea induction but without therapy.

### 2.4. Housing and husbandry

**Figure 1.**
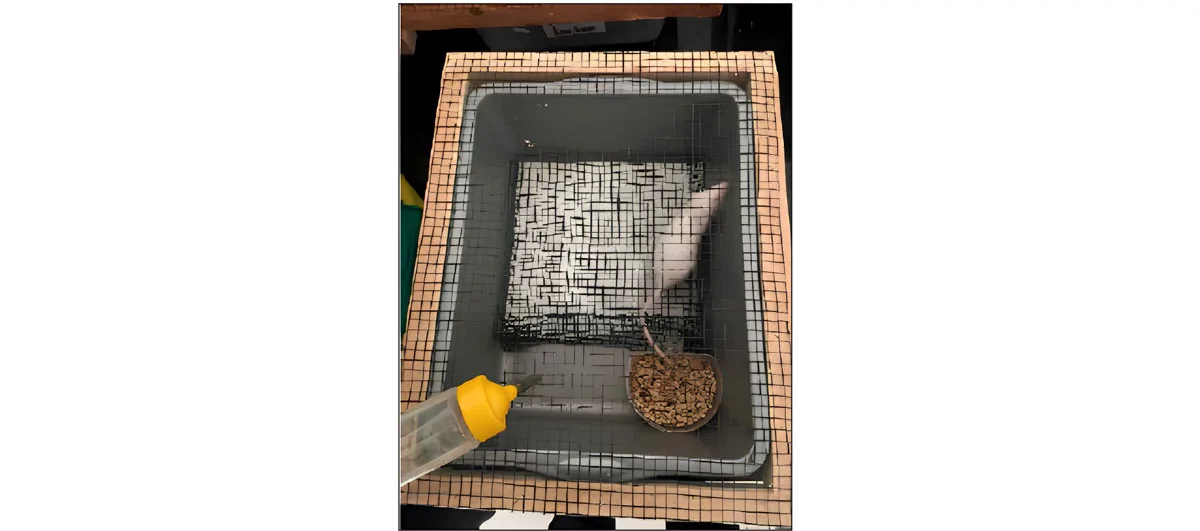
Animal housing.

Experimental mice were housed in standard plastic cages for acclimatization. Appropriate bedding and environmental conditions were controlled (22–25°C, 50–60%RH). Experimental mice were in a 12-h light/dark cycle. Animals had access to standard laboratory food and drinking water ad libitum throughout the study period, except during specific treatment procedures. The mice have wire-mesh bedding to sink their waste after treatment and to collect it. The feces were placed on white bedding to ensure clinical consistency, as shown in Figure 1.

### 2.5. Randomization and allocation

Randomization was performed by the animal house staff of the Faculty of Pharmacy to minimize research bias. Following the acclimatization period, the mice were randomly allocated to five experimental groups (*n* = 5 per group) using simple randomization. The groups consisted of a negative control group comprising healthy mice without diarrhea induction or treatment; a disease control group in which mice were subjected to diarrhea induction without receiving any therapy; a positive control group of mice with diarrhea induction treated with cotrimoxazole; a treatment 1 group in which mice with diarrhea induction received *L. lactis* D4 at a dose of 5 × 10^8^ CFU/mouse in 0.50 ml administered once daily; and a treatment 2 group in which mice with diarrhea induction were treated with *L. lactis* D4 administered twice daily, with each administration containing 5 × 10^8^ CFU/mouse in 0.50 ml.

### 2.6. Induction of diarrhea

Diarrhea was induced by administering a 0.5 ml oral suspension of *S. aureus* (10^9^ CFU/ml) for 3 days until diarrhea symptoms appeared. The consistency of feces was assessed using a three-point scoring system for diarrhea to determine whether the feces were hard or normal, with pellets present. The scale was defined as follows: 1 = hard, normal stool; 2 = soft, full stool. The probiotic dose group received *L. lactis* D4 (10^9^ CFU/ml), administered orally twice daily for 7 days following diarrhea induction, characterized by weight loss, increased fecal weight, and increasingly liquid fecal consistency. Cotrimoxazole was administered as a positive control at 0.40 ml orally for 7 days after diarrhea induction.

### 2.7. Preparation and treatment administration

The *L. lactis* strain D4 isolate used in this study was obtained from the Animal Product Technology Laboratory, Faculty of Animal Husbandry, Andalas University. This isolate was obtained from fermented buffalo milk (Dadiah) in West Sumatra and underwent molecular identification and viability testing to confirm its suitability as a Customized Microbial Supplementation (CMS). The *S. aureus* isolate was obtained from the Animal Product Technology Laboratory at Andalas University.

The choice of 10^9^ CFU/ml in 0.50 ml was based on scientific evidence that bacterial viability in the range of 10^9^ CFU/ml is adequate for colonization of the digestive tract and to produce probiotics consistently. This dose is ideally chosen in animal experiments to induce significant physiological effects such as activation of digestive enzymes, modulation of gut microbiota, and stimulation of the non-specific immune system without influencing the flora.

### 2.8. Outcome measure and data analysis

The observed parameters included: Body weight: measured daily using a digital balance with a precision of 0.01 gm. Fecal consistency: a 3-point scale (1 = hard, 2 = soft, 3 = watery). Fecal weight: Total feces measured using a digital balance with a precision of 0.01 gm. The research data were statistically analyzed using a one-way analysis of variance (ANOVA) in the Statistical Package for the Social Sciences (SPSS). ANOVA was used to assess variation among treatment groups. If the results showed a significant difference, Duncan’s Multiple Range Test (DMRT) was used as a post hoc test to identify which groups differed significantly at the *p* < 0.05 level. The analysis of fecal consistency in this study was viewed using a heatmap. A heatmap of fecal consistency scores across treatment groups is shown in Table 3.

## 3. Results and Discussion

Observation of 25 mice, divided into five groups, showed significant differences (*p* < 0.05) in body weight, fecal consistency, and fecal weight among the treatment groups.

### 3.1. Body weight

ANOVA analysis showed no significant differences from day 0 to 6 (*p* > 0.05). However, from day 7 to 10, significant differences were observed among the groups (*p* < 0.05). The disease control group experienced the greatest weight loss, while probiotic dose 2 exhibited the smallest reduction, with a final mean body weight of 31.04 gm. The body weight profile was provided in Table 1.

**Table 1.** Body weight data (mean ± SD).

Group (mean ± SD)						
Parameter	Negative control	Positive control	Disease control	Treatment 1	Treatment 2	*p*-value
Body weight day 0	28.46 ± 1.82	30.70 ± 1.57	30.94 ± 2.20	28.98 ± 1.88	29.66 ± 2.50	**0.27**
Body weight day 1	29.36 ± 2.43	32.12 ± 1.93	31.70 ± 2.18	29.80 ± 2.21	32.58 ± 1.92	**0.10**
Body weight day 2	29.60 ± 2.25	31.00 ± 1.91	30.40 ± 1.53	28.78 ± 1.64	31.60 ± 1.90	**0.17**
Body weight day 3	30.72 ± 2.31	30.22 ± 2.07	29.38 ± 1.69	28.06 ± 1.19	30.84 ± 2.46	**0.20**
Body weight day 4	30.92 ± 2.45	30.08 ± 2.16	29.12 ± 1.69	27.64 ± 1.33	30.32 ± 2.30	**0.13**
Body weight day 5	31.24 ± 2.40	30.02 ± 2.02	28.66 ± 1.87	28.00 ± 1.70	30.68 ± 2.19	**0.10**
Body weight day 6	31.44 ± 2.40	30.18 ± 2.03	28.06 ± 1.69	28.28 ± 1.85	30.40 ± 2.27	**0.09**
Body weight day 7	31.78 ± 1.77	30.04 ± 2.21	27.96 ± 1.77	28.50 ± 1.81	30.90 ± 1.94	**0.04**
Body weight day 8	32.34 ± 1.92	30.86 ± 2.32	28.08 ± 1.48	29.38 ± 1.43	31.18 ± 2.14	**0.02**
Body weight day 9	32.48 ± 1.69	31.42 ± 2.47	28.92 ± 1.60	29.80 ± 1.28	31.58 ± 2.16	**0.05**
Body weight day 10	32.64 ± 1.47	31.84 ± 1.96	28.84 ± 0.94	30.60 ± 1.56	31.80 ± 2.02	0.01*

Note: (*) one-way ANOVA test results (*p* < 0.05).

### 3.2. Fecal weight

ANOVAs of fecal weight showed highly significant differences among groups on observation days from the start of treatment (*p* < 0.05). The disease control group had the highest fecal output, whereas probiotic dose 2 showed the greatest reduction, with values comparable to those of the negative control, as shown in Table 2.

**Table 2.** Fecal weight data (mean ± SD).

Group (mean ± SD)						
Parameter	Negative control	Positive control	Disease control	Treatment 1	Treatment 2	***p***-value
Fecal weight day 0	0.75 ± 0.00^c^	0.69 ± 0.00^e^	0.74 ± 0.00^a^	0.70 ± 0.00^b^	0.74 ± 0.00^d^	0.00*
Fecal weight day 1	0.69 ± 0.00^d^	0.91 ± 0.00^a^	0.75 ± 0.00^c^	0.58 ± 0.00^e^	0.90 ± 0.00^b^	0.00*
Fecal weight day 2	0.54 ± 0.00^e^	0.94 ± 0.00^a^	10.9 ± 0.00^b^	1.00 ± 0.00^d^	0.96 ± 0.00^c^	0.00*
Fecal weight day 3	0.52 ± 0.00^e^	1.00 ± 0.00^a^	1.09 ± 0.00^d^	0.98 ± 0.00^b^	0.90 ± 0.00^c^	0.00*
Fecal weight day 4	0.51 ± 0.00^e^	1.22 ± 0.00^a^	1.24 ± 0.00^d^	0.79 ± 0.00^c^	0.99 ± 0.00^b^	0.00*
Fecal weight day 5	0.27 ± 0.00^e^	0.82 ± 0.00^a^	0.52 ± 0.00^d^	0.81 ± 0.00^c^	0.54 ± 0.00^b^	0.00*
Fecal weight day 6	0.45 ± 0.00^e^	0.59 ± 0.00^a^	1.25 ± 0.00^c^	0.80 ± .000^d^	0.55 ± 0.00^b^	0.00*
Fecal weight day 7	0.31 ± 0.00^e^	0.58 ± 0.00^d^	1.27 ± 0.00^b^	0.50 ± 0.00^a^	0.46 ± 0.00^c^	0.00*
Fecal weight day 8	0.35 ± 0.00^e^	0.55 ± 0.00^d^	1.23 ± 0.00^c^	0.28 ± 0.00^a^	0.36 ± 0.00^b^	0.00*
Fecal weight day 9	0.33 ± 0.00^e^	0.50 ± 0.00^c^	1.19 ± 0.00^b^	0.27 ± 0.00^a^	0.33 ± 0.00^d^	0.00*
Fecal weight day 10	0.27 ± 0.00^e^	0.33 ± 0.00^d^	0.87 ± 0.00^c^	0.27 ± 0.00^a^	0.30 ± 0.00^b^	0.00*

Note: (*) One-way ANOVA test results (*p* < 0.05) (^a, b, c, d, e^): data with different superscripts indicate significant differences in Duncan’s advanced test results (*p* < 0.05).

### 3.3. Fecal consistency

**Figure 2.**
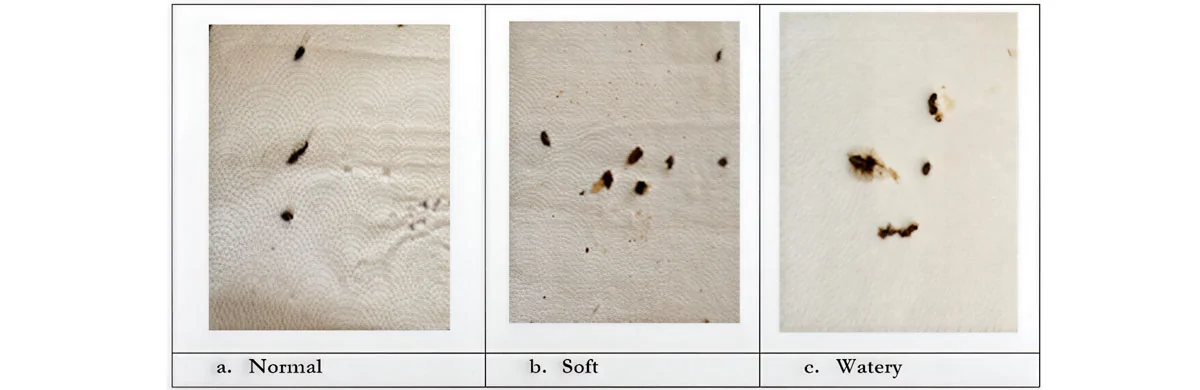
Fecal consistency.

Fecal consistency observations in Table 3. showed distinct variations among groups. In the disease control group, the feces remained mostly stable (soft to watery) all through the observation period. The negative control group, always in the hard area, scored 1, but cotrimoxazole was able to normalize the stool as soon as day 6 started. Probiotic-treated groups were experiencing earlier improvements because dose 1 was becoming stable on day 5. Dose 2 became almost normal (score 1) by day 4 and remained that way until the final time of the study. Figure 2 shows various types of fecal consistency.

**Table 3.** Fecal consistency scores.

Mice	Day 0	Day 1	Day 2	Day 3	Day 4	Day 5	Day 6	Day 7	Day 8	Day 9	Day 10
Disease	1	1	2	3	3	2	3	3	2	2	2
Disease	1	1	2	3	3	2	3	3	2	2	2
Disease	1	1	2	3	3	2	3	3	2	2	2
Disease	1	1	2	3	3	2	3	3	2	2	2
Disease	1	1	2	3	3	2	3	3	2	2	2
Negative	1	1	1	2	1	2	1	1	1	1	1
Negative	1	1	1	2	1	2	1	1	1	1	1
Negative	1	1	1	2	1	2	1	1	1	1	1
Negative	1	1	1	2	1	2	1	1	1	1	1
Negative	1	1	1	2	1	2	1	1	1	1	1
Positive	1	1	2	3	3	2	1	1	1	1	1
Positive	1	1	2	3	3	2	1	1	1	1	1
Positive	1	1	2	3	3	2	1	1	1	1	1
Positive	1	1	2	3	3	2	1	1	1	1	1
Positive	1	1	2	3	3	2	1	1	1	1	1
Treatment 1	1	1	2	3	3	2	2	1	1	1	1
Treatment 1	1	1	2	3	3	2	2	1	1	1	1
Treatment 1	1	1	2	3	3	2	2	1	1	1	1
Treatment 1	1	1	2	3	3	2	2	1	1	1	1
Treatment 1	1	1	2	3	3	2	2	1	1	1	1
Treatment 2	1	1	3	3	3	1	2	1	1	1	1
Treatment 2	1	1	3	3	3	1	2	1	1	1	1
Treatment 2	1	1	3	3	3	1	2	1	1	1	1
Treatment 2	1	1	3	3	3	1	2	1	1	1	1
Treatment 2	1	1	3	3	3	1	2	1	1	1	1

Notes: 1 = hard, 2 = soft, 3 = watery

The results of this study show that administration of the probiotic *L. lactis* D4 seems to prevent diarrhea [16]. In general, there were significant differences in body weight from day 7 to day 10 when subjects received a probiotic. The progression of body weight between feeding groups may be indicative of differences in growth in the treatment groups during this procedure. This study indicates that the probiotic colonization process takes weeks to minimize the amount of *S. aureus* in an environment and maintain microbial equilibrium there [17, 18].

For fecal weight, significant differences between groups were detected as early as day 1 of observation, with the probiotic groups consistently showing reductions relative to the other groups. This result indicates that *L. lactis* D4 rapidly suppresses excessive intestinal fluid secretion. Consistent with a previous report, these results showed that *L. lactis* produces lactisin with potent antimicrobial activity against *S. aureus* [19]. The rapid response indicates that *L. lactis* D4 modulates intestinal physiology early by suppressing excessive intestinal fluid secretion and mitigating inflammation. This result is consistent with previous reports showing that *L. lactis* produces bacteriocins, such as lactisin, which have potent antimicrobial activity against *S. aureus* and other enteric pathogens [20, 21, 22].

Fecal consistency has been shown to follow a similar trend. The disease control group maintained scores of 2–3 (soft to watery) throughout the study, whereas the probiotic-treated groups showed earlier improvements than those treated with cotrimoxazole [23]. The probiotic group at dose 2 reached near-normal stool consistency (score 1) on day 4, which occurred more quickly than dose 1 (day 5) or cotrimoxazole (day 6), indicating that probiotics work to control the production of disease-causing bacteria and to restore health in the gut in combination with beneficial bacteria. We have also shown that probiotics are useful in regulating the immune system, in particular, in reducing inflammation and enhancing the body’s immune response, thereby improving recovery from digestive tract conditions [7, 24, 25, 26].

The promising effects of *L. lactis* D4, compared with cotrimoxazole, are significant. Although antibiotics effectively eradicate pathogens, they can also disrupt the commensal microbiota and delay the restoration of gut homeostasis. [27]. Probiotics have direct antimicrobial activity and restoration of microbial diversity. *Lactococcus lactis* strains produce short-chain fatty acids (SCFAs), particularly acetate and lactate, which reduce intestinal pH, inhibit pathogen proliferation, and serve as an energy source for colonocytes [28].

The increase in the size of the error bars at the end of the study indicates differences in the growth of the experimental animals. Some of them are natural variations among individuals and differences in their ability to utilize feed, as well as environmental influences such as housing conditions. Such differences can affect the uniformity of the animals and would also contribute to the treatment results.

In general, this study suggests that *L. lactis* D4 has therapeutic potential as an adjunct or alternative to conventional antibiotics for the management of bacterial diarrhea. The rapid onset of action in improving fecal parameters, combined with its capacity to promote intestinal homeostasis, shows the clinical promise. Furthermore, this research contributes to the achievement of the Sustainable Development Goals (SDGs), particularly SDG 3 (Good Health and Well-being) through the development of therapeutic materials. The use of these probiotics can support improved gastrointestinal health and nutrient absorption in the context of zero hunger (SDG 2). This research also supports SDG 12 (Responsible Consumption and Production) by encouraging the use of natural ingredients in therapeutic approaches.

### 3.4. Strengths and limitations

This study has the advantage of using *L. lactis* D4 from Dadiah as a local probiotic and comparing it directly with antibiotics. This provides clear evidence that probiotics have the potential to serve as an alternative treatment for diarrhea. However, this study has limitations, as it was conducted only on experimental animals and over a relatively short observation period. Therefore, its long-term effects on humans remain unknown. Further human studies are needed to confirm its benefits.

## 4. Conclusions

In conclusion, oral administration of CMS *Lactococcus lactis* D4 tended to improve growth performance during the early feeding phases; however, the differences between the CMS and control groups were less pronounced in the final phase, indicating that further studies are required to confirm the long-term impact of the intervention. However, further clinical trials are required to confirm its efficacy in humans.

## Data Availability

The data presented in this study are available from the corresponding author upon reasonable request.

## List of abbreviations

ANOVA, analysis of variance; ARRIVE, Animal Research: Reporting of In Vivo Experiments; CFU, colony-forming unit; CMS, customized microbial supplementation; DMRT, Duncan’s multiple range test; LAB, lactic acid bacteria; LD4, *Lactococcus lactis* D4; ml, milliliter; gm, gram; CFU/ml, colony-forming unit per milliliter; MRSA, methicillin-resistant *Staphylococcus aureus*; SCFA, short-chain fatty acids; SPSS, statistical product and service solutions; WHO, World Health Organization.
